# The use of antidementia therapy in non-dementia cognitive disorders associated with COVID-19

**DOI:** 10.1192/j.eurpsy.2023.1704

**Published:** 2023-07-19

**Authors:** P. Liubov, I. Iashina

**Affiliations:** 1 Department of gerontopsychiatry; 2 Moscow Research Institute of Psychiatry - branch of the V. Serbsky National Medical Research Center for Psychiatry and Narcology, Moscow, Russia, Moscow, Russian Federation

## Abstract

**Introduction:**

The new coronavirus infection causes severe damage to the human body. One of the most serious complication is cognitive impairment.

According to a meta-analysis by Maxime Taquet *et al.* (2021), which assessed neurological and psychiatric outcomes among 236,379 patients diagnosed with COVID-19, 1.7% of participants over 65 years of age were diagnosed with dementia.

In a US online survey of 1,500 people (Sarah Ballou *et al.*, 2020), about half reported difficulty concentrating on any task after experiencing COVID-19.

It was also found that there is a decrease in the speed of reactions and problem-solving (Jeffrey D. Pyne *et al*., 2021).

There are a number of studies that present the evidence-based efficacy of acetylcholinesterase (AChE) inhibitors in dementia prevention.

However, in accordance with clinical guidelines for cognitive disorders in the elderly, anti-dementia drugs are not used at the stage of mild to moderate cognitive impairment (Ministry of Health of the Russian Federation, 2020).

**Objectives:**

The use of AChE inhibitors in cognitive impairments that do not reach the degree of dementia.

**Methods:**

Research data from open sources.

**Results:**

The development of early cholinergic deficiency correlates with the development of cognitive impairment, while acetylcholine has a pronounced neuroplastic effect and increases the number of neurons (Gabriela Dumitrita Stanciu *et al*., 2019).

In a double-blind, placebo-controlled study, a positive effect of rivastigmine was found in patients with mild to moderate cognitive impairment. The study’s results show that rivastigmine treatment (3, 6, 9 mg/day) for six months increases brain activity of the hippocampus in the control group by 32,5%. Rivastigmine prevented the clinical progression of symptoms of cognitive impairment and caused activation of some parts of the cerebral cortex (Nagaendran Kandiah *et al.*, 2017).

In a study by Wolfson C. *et al.* (2002), it was found that rivastigmine can slow down the development of cognitive impairment for at least six months in patients with mild to moderate massive cognitive dysfunction. Subjects treated with 1 to 21 mg per day for 7 to 12 weeks got more favorable ADAS-cog scores for the six months after treatment. While those who took the drug in doses of 6 to 12 mg showed a more pronounced positive effect compared to the placebo group.

The Luca Rozzini in 2006 conducted a study based on 59 subjects with mild cognitive impairment. 15 subjects received both neuropsychological examination and acetylcholinesterase inhibitors. As a result, the remaining subjects were behind in terms of abstract thinking and behavioral symptoms, in comparison with a combined treatment group.

**Conclusions:**

It is advisable to conduct further studies on the effectiveness of AChE inhibitors to prevent the progression of mild to moderate cognitive impairment and their transition to dementia.

**Disclosure of Interest:**

None Declared

